# Spasm in Chronic Nerve Disease: Being the Gulstonian Lectuers for 1886

**Published:** 1886-12

**Authors:** 


*Spasm in Chronic Nerve Disease: being the Gulstonian Lectuers for* 1886. By SEYMOUR J. SHARKEY, M.A., M.B. Oxon., F.R.C.P. London: J. & A. CHURCHILL. 1886.

Dr. Sharkey has been well advised in reprinting in book-form his Gulstonian lectures for the present year, for they are too valuable to be allowed to be nothing more than a mere ingredient in the *olla podrida* which forms the contents of our weekly journals. In treating of spasm in chronic nerve disease, Dr. Sharkey divides the subject into these divisions, viz:—(1) spasm in connection with cerebral motor mechanism; (2) in connection with spinal mechanism; (3) functional spasm. Considering that our present knowledge of anatomy, physiology, and pathology, does not justify us in concluding that there is any efferent motor connection between the brain and the spinal cord except the pyramidal tract, direct and crossed, Dr. Sharkey adduces evidence and arguments to show that it is disease of the pyramidal tract *alone* which gives rise to chronic muscular spasm: this is the more interesting, for, although everyone is agreed that the pyramidal tracts are the most frequent agents in producing spasm, Hughlings Jackson and many other neurologists believe in the existence of "cerebellar rigidity," due to unantagonised cerebral influx. Dr. Sharkey, however, maintains that the cerebellum *per se* has no part in the production of spasm; and that a tumour of the cerebellum does not produce contractures, unless it be in such a position and of such size and substance as to produce pressure on the pons varolii or medulla oblongata, and so press upon some portion of the pyramidal tracts which lie therein. Cases are also quoted to prove that destruction of the corpora striata and optic thalami does not give rise to spasm, so long as the internal capsule remains intact.

In speaking of spasm in connection with spinal mechanisms, Dr. Sharkey concludes that muscular spasm is rarely caused directly by chronic disease of peripheral motor nerves, the distortions often seen in such cases being brought about by the unopposed action of the unaffected muscles. Spasm produced in a reflex manner by disease of afferent nerves can hardly be considered apart from spasm produced by disease of ganglionic cells; for it is doubtful whether stimuli applied to afferent nerves would produce muscular spasm if the centres were healthy. There does not seem to be any evidence that gross disease of ganglionic cells produces spasm; though impalpable disorders do, such as those resulting from their separation from their cerebral centres.

Upon that subject of confessed ignorance, functional spasm, Dr. Sharkey's remarks are philosophical, interesting, and instructive. He points out that functional disorders of the cerebral motor mechanism produce spasmodic conditions resembling those from gross disease. As in cases of congenital absence of portions of the motor centres of the cortex with correlated non-development of the corresponding portion of the pyramidal tract there are found spastic conditions like those occurring in hemiplegia, it is evident that the mere absence of voluntary motor impulse is enough to give rise to the phenomena in question; and it is legitimate, therefore, to conclude that suppression of the functions of this tract is the cause of functional hemi-spasm. We cannot give space to notice the suggestions which Dr. Sharkey has to make upon other classes of functional spasmodic affections, such as writers' cramp and other "professional hyperkineses," "latent contracture," and so on; but can only conclude by heartily recommending the work as well worthy of being carefully studied, and as reflecting the highest credit upon its author.

